# Super-Resolution solid-state NMR Spectroscopy

**DOI:** 10.1007/s10858-026-00490-5

**Published:** 2026-03-31

**Authors:** Olivia Gampp, Riccardo Cadalbert, Roland Riek, Sarah A. Overall

**Affiliations:** Institute of Molecular Physical Science, ETH Zürich, Vladimir-Prelog-Weg 2, Zürich, CH-8093 Switzerland

## Abstract

Solid-state NMR spectroscopy is often limited by low spectral resolution, a problem typically addressed using fast magic-angle spinning (MAS) and ¹H detection, which require costly specialized hardware. Here, we demonstrate that the super-resolution method—previously applied in solution-state NMR—can be successfully implemented in solid-state NMR to enhance resolution. Applying dynamic number of scans (DNS) sampling to 2D ¹³C-¹³C DARR experiments on the AP205 capsid protein yielded an effective doubling of resolution, halving peak widths from ~ 180 Hz to ~ 87 Hz. Furthermore, DNS acquisition provides a significant advantage over post-acquisition apodization of conventional data with a 20% gain in sensitivity, yielding 309 more detectable peaks with 20% more sequential contacts and 25% more long-range contacts. This method is simple to implement and provides a powerful, accessible strategy to greatly improve the quality of solid-state NMR spectra applicable at all MAS frequencies.

**Supplementary Information:**

The online version contains supplementary material available at 10.1007/s10858-026-00490-5.

## Introduction

Although structure determination has become a routine procedure in solution-state NMR spectroscopy (Berndt et al. 1992, Wüthrich 1990, Kaptein et al. 1985, Güntert 2009), *de novo* structure determination by solid-state NMR remains challenging (Castellani et al. 2002, Barbet-Massin et al. 2014). This is largely due to the limited spectral resolution caused by strong homonuclear dipolar interactions and heterogeneous line broadening. Significant gains have been obtained through ultra-fast spinning methodologies at magic-angle spinning (MAS) frequencies of up to 160 kHz, which average the homogeneous contributions to the linewidth to first order, enabling ^1^H detection and providing a net sensitivity boost of 8 fold (Sun et al. 2025). The resolution gains of ultra-fast MAS provide the necessary resolution to obtain ^1^H side-chain assignments and contacts, information critical for *de novo* structure determination (Callon et al. 2023). It has been predicted that MAS frequencies of 300 kHz will be required for maximum ^1^H resolution for many biomolecular samples not in the rigid limit (Xue et al. 2018). However, strong ^1^H-^1^H dipolar coupling networks are still a limiting factor for the resolution of ^1^H detected solid-state spectra for large proteins. Selective deuteration greatly dilutes this ^1^H dipolar coupled network, improving spectral resolution by reducing homogeneous coherent contributions to the linewidth, even under ultra-fast MAS (Xue et al. 2017). Together, increasing external magnetic field strength, ^1^H dilution through deuteration and ultra-fast MAS form the state-of-the-art pillars of high resolution biomolecular structural studies by solid-state NMR. However, the benefit of these techniques is diminished in heterogeneous samples whose linewidth have large contributions from inhomogeneous broadening (Marchand et al. 2022).

The homogeneous contribution to the linewidth can be further reduced through analytical methods which deconvolute the homogeneous contribution either through acquisition of multiple data sets at multiple MAS frequencies (Cordova et al. 2023, Moutzouri et al. 2021) or through hahn-echo deconvolution (Perras 2024) to predict and then remove this contribution from the spectrum. These methods achieve significant and impressive increases in ^1^H linewidths of standard crystalline amino acids (Moutzouri et al. 2021, Moutzouri et al. 2023) or proteins such as GB1 (Perras et al. 2025) both at ultra-fast and slower MAS frequencies yielding resolution enhancements of over 7 and 2 respectively. However, these methods require the measurement of multiple datasets (Moutzouri et al. 2021), substantial post-processing (Manu et al. 2019) or trained machine learning algorithms to achieve.

At slower spinning speeds, the resolution of ^13^C detected experiments can be enhanced through sparse ^13^C labeling (Liu et al. 2016) or deuteration of the protein backbone (Tang et al. 2010), reducing the coherent contribution to the ^13^C linewidth by 1.5 and 2 respectively. In fact, such methods have also demonstrated significant improvements even in ^1^H detected spectra under ultra-fast MAS (Zhou and Rienstra 2008, Xue et al. 2017) achieving resolution enhancements of 3-fold.

Recently, the super-resolution method was applied to 2D homonuclear experiments in solution-state NMR, achieving a halving of the peak-width in the indirect dimension (Gampp et al. 2024, Wenchel et al. 2024). This technique uses a dynamic number of scans (DNS) acquisition scheme (Waudby and Christodoulou 2020, Simon and Köstler 2019), weighting the scan count per t₁ increment by a combined exponential-cosine function (Eq. 1) to counteract signal decay and enhance resolution (Gampp et al. 2024, Wenchel et al. 2024).

1 $${e}^{{R}_{enh}{t}_{1}}cos\left(\frac{\pi{t}_{1}}{2{t}_{max}}\right)$$

Where *R*_*enh*_ *= R*_*2*_ *(1- f), *R*_*2*_ is the estimated transverse relaxation of the sample and *f* the reduction factor, which is chosen as 0.5 to reduce the peak width by 50%. While the linewidth is dominated by homogeneous contributions in solution-state, due to dynamic motion, its application to solid-state NMR should also be beneficial despite the additional large heterogeneous contributions.

Here, we apply this super-resolution approach to solid-state NMR. We demonstrate its efficacy on the model protein ubiquitin and the challenging non-crystalline 25 kDa homodimeric AP205 capsid protein. We show that the method improves the resolution by a factor 2 and yields a sensitivity advantage over similar post-acquisition processing methods (Levitt et al. 1984), resulting in a significant increase of usable peaks and long-range restraints for structure calculation. The method is easy to implement into any pulse program and requires minimal post-processing outside of the TopSpin interface, making its implementation accessible to a broad range of user expertise.

## Results

### The super-resolution technique enhances resolution in homonuclear 2D solid-state NMR spectra

To evaluate the application of dynamic number of scans DNS sampling to solid-state NMR, we acquired a 2D [¹³C,¹³C]-DARR spectrum of microcrystalline U-^13^C,^15^N-Ubiquitin with and without the super-resolution sampling scheme. DNS acquisition modulates the scan count per t₁ increment with a combined exponential-cosine weighting function to counteract signal decay as describe by Wenchel et al. (Wenchel et al. 2024). and Gampp et al. (Gampp et al. 2024). (see Methods). An estimate of T_2_* is required to calculate the optimal experimental parameters and is determined from the peak width (FWHH) of a resolved peak. This can be done in 1D if resolution permits, however it is optimal to run a short 2D-DARR and measure the linewidth of a resolved peak in the indirect dimension (**SI-1**). We could obtain good estimates of the average linewidth from a 1.5 h DARR. A variable counter list is then generated using Eq. 1, executed by a python script (**SI-2**), the generated list is then utilized by TopSpin.

**Fig. 1:**
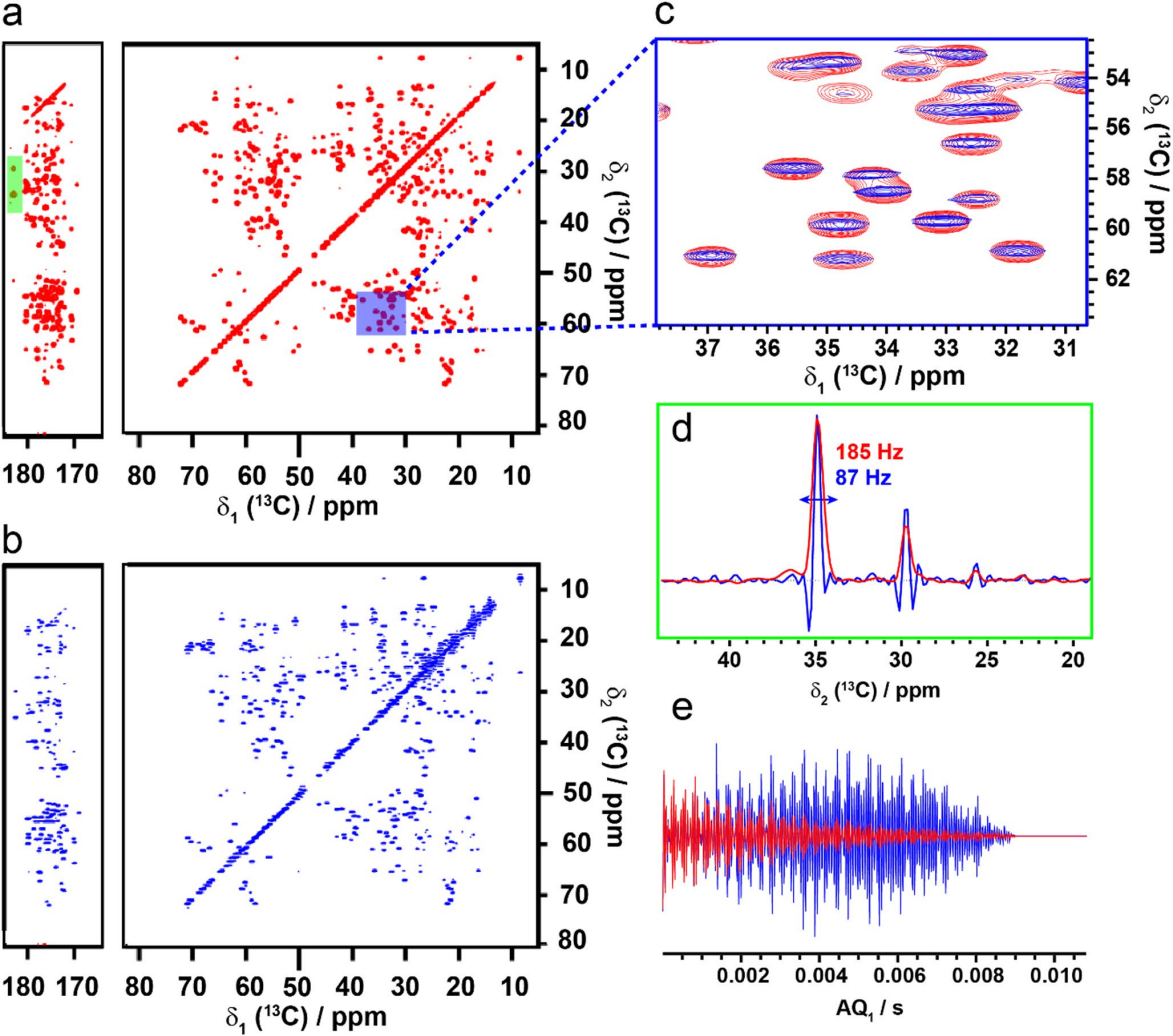
Implementation of super-resolution spectroscopy in the solid-state. (a) 20 ms conventional ^13^C-^13^C DARR spectrum of microcrystalline U-^13^C,^15^N-Ubiquitin acquired with 28 scans, 1216 increments in the indirect dimension and 2048 points in the direct dimension. (b) Super-resolution enhanced ^13^C-^13^C-DARR spectrum of microcrystalline U-^13^C,^15^N-Ubiquitin acquired with 4 scans (28 scans average over the full acquisition), 1216 increments in the indirect dimension and 2048 points in the direct dimension. Where R_2_ = 1050 Hz (334 Hz width) was used to generate the sampling schedule and number of increments in the indirect dimension, where R_2_ = π(FWHH). The FID is additionally processed with a smoothing function prior to Fourier transform. (c) Overlay of the conventionally acquired spectrum (red) and the super-resolution enhanced spectrum (blue) of the region indicated by the blue shaded area in (a). (d) Comparative linewidth of the peaks in the green shaded area in (a). (e) FID of the first increment of the conventional (red) and super resolution (blue) DARR spectra. Data was acquired at 17 kHz MAS at 850 MHz ^1^H frequency at 275 K

The full implementation of DNS acquisition in the DARR experiment is given in **SI-3**. With this implementation, the SR-DARR runs for an experiment time equivalent to a 28 scan per increment experiment when starting with 4 scans in the first increment and having a phase cycle of 4. The step size is therefore dictated by the minimum phase cycle. Comparison of the SR-DARR with the conventionally acquired DARR on microcrystalline U-^13^C,^15^N-Ubiquitin demonstrates a factor 2 reduction in peak width when the same number of increments in the indirect dimension are acquired (Fig. 1a-c**)** thus the resolution is increased per unit time. The apparent linewidth is reduced from approximately 185 Hz to 87 Hz (Fig. 1d), representing an effective doubling of the resolution and confirming the successful transfer of this method from solution-state to solid-state NMR.

Inaccuracies in the estimation of T_2_* can be relatively large before the efficiency of the method is reduced or the quality of the spectrum is degraded. A high quality spectrum is readily produced even if the R_2_ estimate is 2 times that of the actual peak width (**SI-4a**). Overestimating the linewidth, and therefore R_2_, is the most likely error to occur in solids and results in the appearance of sinc function oscillations in peaks for which the actual R_2_ is much smaller than the estimate used to generate the variable counter list (**SI-4b**). Much of the artifacts generated in this case can be compensated for with a quadratic sine (qsine) function during processing. However, if the spectrum contains a large range of R_2_ values, application of a qsine function can be problematic. If the estimated R_2_ (R_2,est_) is 2 times smaller than the true R_2_ (R_2,true_) i.e.: R_2,est_ = 1/2R_2,true_, qsine correction may reduce the peak intensity below the limit of detection (LOD), due to amplification of noise at later increments (**SI-5**). If R_2,est_ > R_2,true_ then qsine correction broadens the peak width. Beyond R_2,est_ = 4R_2,true_, qsine correction no longer fully compensates for fid truncation and residual sinc wiggles are still observed in the spectrum.

Given the large tolerance of the experiment to reasonable errors in R_2_ estimations, such artifacts should not normally occur (**SI-5**). Overestimating R_2_ by two fold retains the factor 2 improvement in resolution. Beyond this, the resolution gain decreases to 1.4 and 1.3 when the overestimate is 3 and 4 times the true R_2_ value respectively. In this case the spectrum becomes truncated if insufficient increments are acquired (as the number of increments and therefore the scaling of the number of scans per increment are determined from the R_2_ estimate). On the other hand, underestimating the linewidth results in the benefit of the DNS scheme being reduced to 1 when R_2,est_ = 1/2R_2,true_. The sensitivity losses in this case will also be increased as small R_2_ estimates will result in a slow initial ramping of the number of scans. Therefore, much of the FID decays before the number of scans becomes large, amplifying the noise. We could obtain R_2_ estimates well within our margin of error from both a 1D spectrum and a short 2D DARR (**SI-1**). We therefore recommend that the appropriate R_2_ is that which represents the average peak width or the value in which most peaks are within 3R_2,est_ > 0.5R_2,est_. Such a spectrum does not require qsine correction and thus retains maximum resolution gains and SNR across all peaks.

The data can be processed normally in TopSpin, without the application of any window function to the indirect dimension as the application of DNS does not alter the FIDs envelope if R_2_ is chosen correctly. Although small artifacts in the FID appear due to the discontinuous step in the number of scans per increment (SI-6) causing side-lobes to be pronounced on intense peaks, particularly on the diagonal (**SI-6)**.

In this case a smoothing function, which corrects for the discrete nature of the number of scans can be applied. The smoothing window function is determined by the continuous number of scans NS_cont_ that should have been acquired for that increment divided by the discrete number that was feasible to acquire given the phase cycle. An example of the smoothing function written in PROSA (Güntert et al. 1992) or python is given in (**SI-7**) and SI-8 respectively. For homonuclear correlation experiments with strong diagonals, smoothing is necessary to obtain the maximum SNR of the SR spectrum. This is due to discretization of the FID caused by scan increment being an integer multiple of the phase cycle (Gampp et al. 2024). Alternatively, increasing the starting number of scans while maintaining a minimum scan step size will render the smoothing function unnecessary. For heteronuclear correlation experiments, these effects are minimal and the smoothing function becomes optional. In addition, the current implementation of DNS limits the indirect acquisition time to ~ 14 ms, for uniformly labeled samples, due to evolution of the J_CC_ coupling during acquisition, which will cause the peaks to start splitting, reducing the resolution and distorting peak shapes. This is a limiting factor for samples which have very long T_2_* relaxation rates, as is the case for microcrystalline ubiquitin. We limited the indirect acquisition time to 9.1 ms (J_COCα_) due to decoupling limitations of the probe. In this case, the value of R_2_ for the increment calculation becomes 1050 Hz rather than the 581 Hz estimated from the linewidth. For such a sample, with very high resolution in the conventional spectrum, (Fig. 1**)** the benefit of the super resolution method for structural studies is not immediately clear as this spectrum is not resolution limited.

**Fig. 2:**
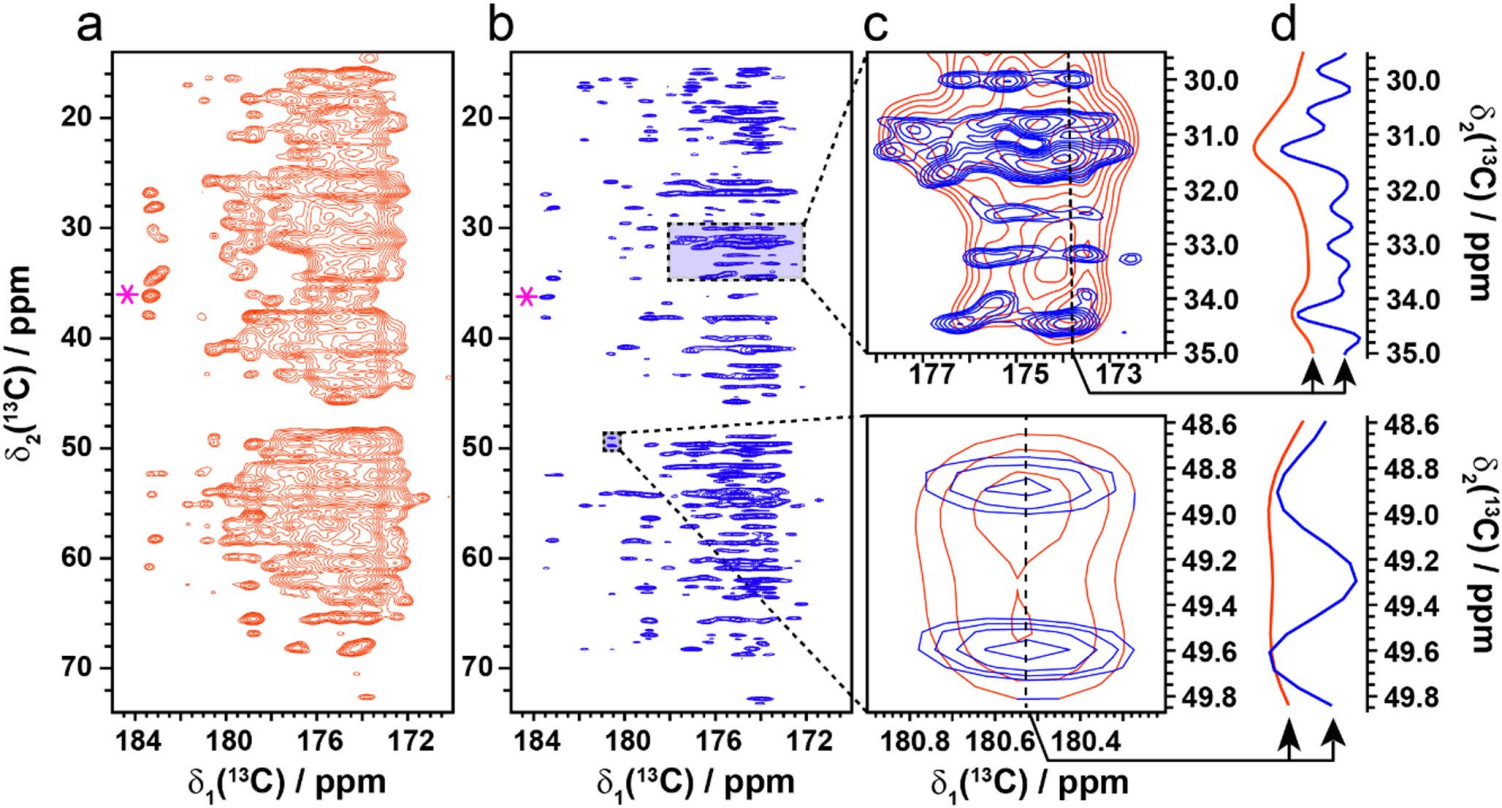
Super-resolution enhancement of non-crystalline U-^13^C,^15^N-AP205 particles. (a) The carbonyl region of a conventionally acquired [^13^C,^13^C]-DARR spectrum. The peak marked with a pink asterisk has a full width half height (FWHH) of 180 Hz. (b) The carbonyl region of the SR-[^13^C,^13^C]-DARR spectrum. The spectrum was measured with an R_2_ = 1050 Hz (334 Hz width). The spectrum was additionally processed with a smoothing window function applied to the indirect FID prior to Fourier transform. The FWHH of the peak marked with an asterisk is 80 Hz. (c) Enlarged regions from the SR-[^13^C,^13^C]-DARR (blue) overlayed with the conventionally acquired spectrum (red). (d) Cross sections through the indirect dimension along the dotted line shown in (c). All spectra were acquired with an average number of scans of 28 and 1216 indirect increments at 850 MHz ^1^H frequency with an MAS frequency of 20 kHz and 500 ms DARR mixing at 275 K

In order to demonstrate the full potential of super-resolution spectroscopy to facilitate structural biology in the solid-state, we acquired a super resolution spectrum of non-crystalline U-^13^C,^15^N-AP205 coat protein, a challenging 25 kDa homodimeric system that additionally assembles into large capsid particles of T = 3 quasi-equivalent icosahedral symmetry (Meng et al. 2024, Shishovs et al. 2016). Even in this heterogeneous system, the spectral resolution was greatly increased with the apparent linewidth reduced from approximately 180 Hz to 80 Hz (Fig. 2a-b), yielding exceptional resolution, particularly in the crowded 500 ms SR-DARR spectrum. The potential for improving the extraction of long-range information for structural studies even in 2D spectra is apparent.

**Fig. 3:**
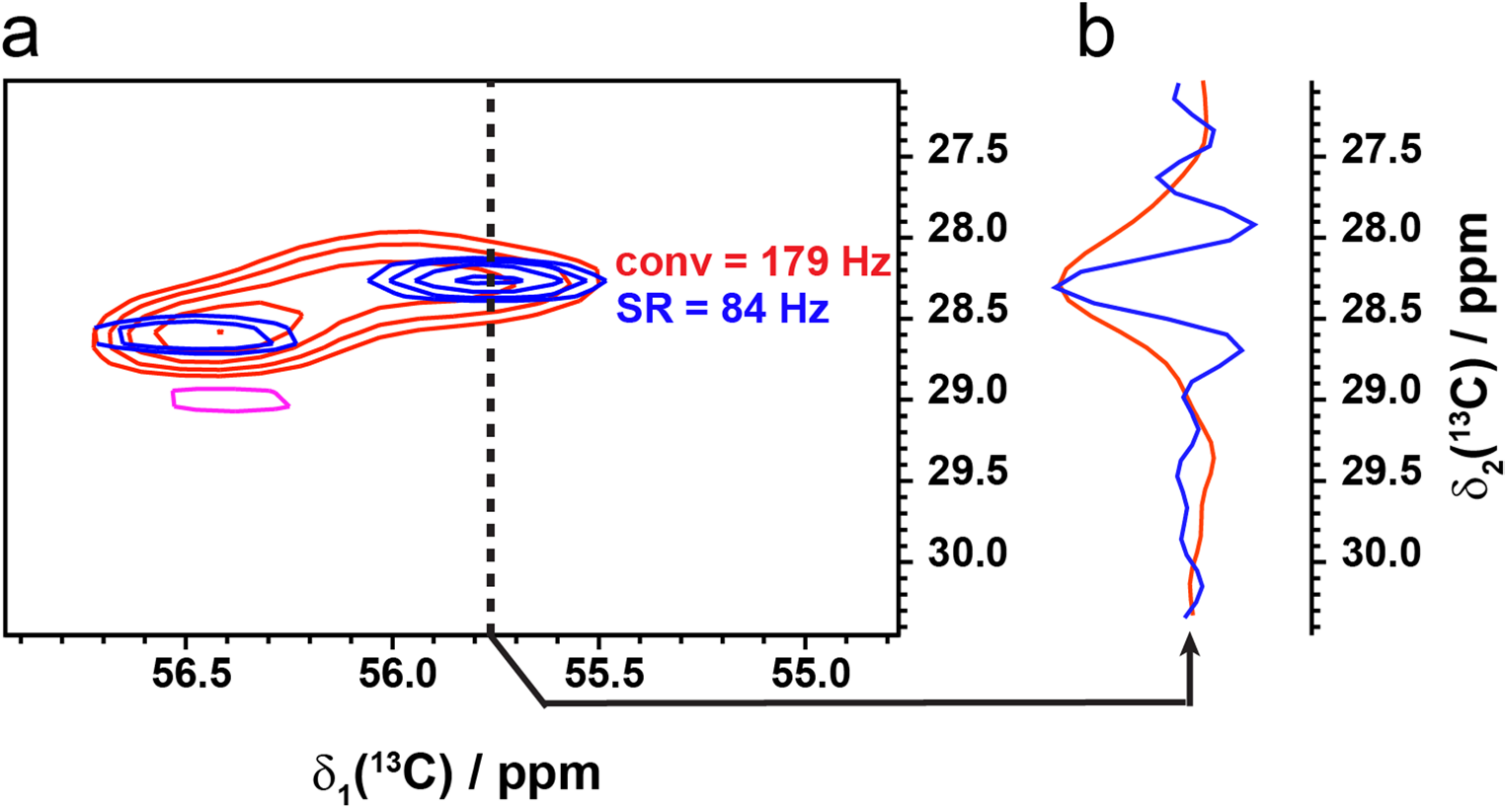
Resolution enhancement is MAS independent. (a) hCH spectrum of conventionally acquired U-^13^C,^15^N-AP205 (red) and DNS sampled spectra (blue) acquired at 50 kHz MAS and 1.2 GHz ^1^H frequency with an R_2_ = 1050 Hz (334 Hz width). (b) 1D slice through the ^13^C indirect dimension showing the linewidth of the conventionally acquired spectrum (red) and the DNS sampled spectrum (blue). Linewidths are determined as the full width at half height (FWHH) of the peaks shown

**Fig. 4:**
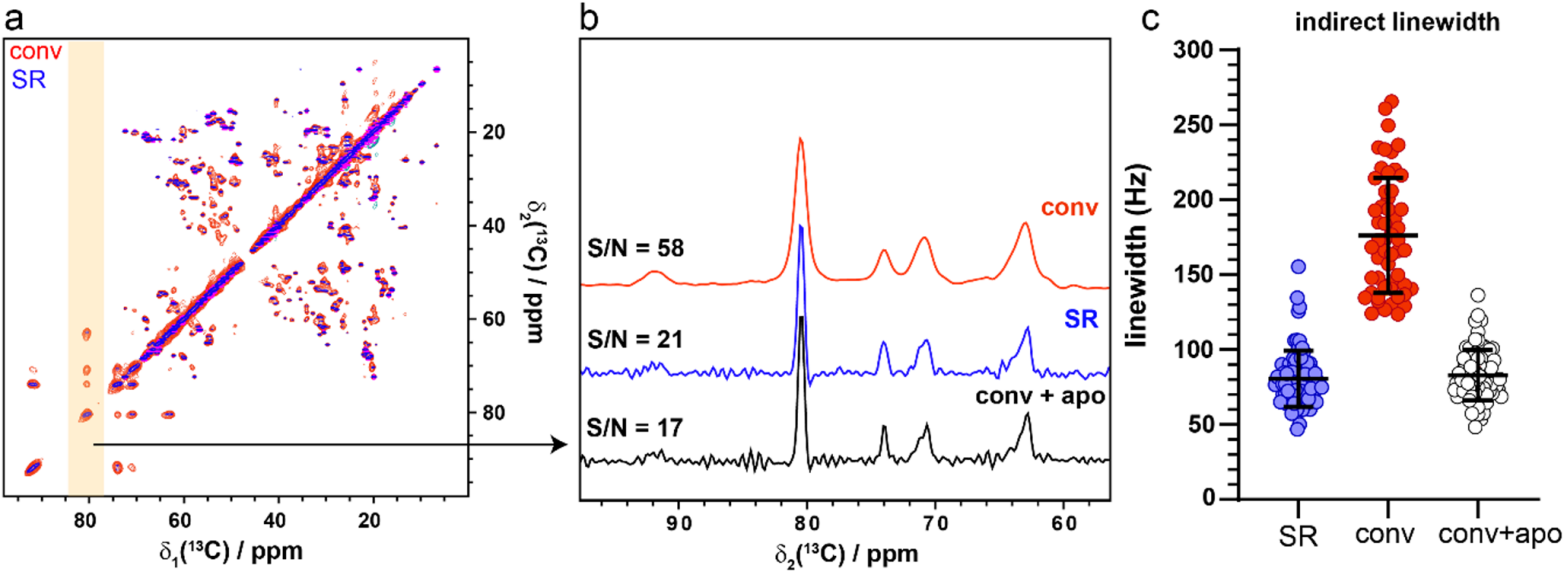
Comparison of spectrum sensitivity and resolution gain of SR-DARR with post-acquisition apodization. (a) Aliphatic region of [^13^C,^13^C]-DARR of U-^13^C,^15^N-AP205 acquired at 850 MHz with 20 kHz MAS and 20 ms homonuclear mixing. The conventional (conv) spectrum (red) is overlayed with the super-resolution version (blue), both spectra were acquired for 29 h and shows the signal-to-noise loss of approximately a factor of 3. (b) Cross sections of the peaks in the orange shaded region in (a) along the indirect dimension showing the conventionally acquired spectrum (red), super resolution spectrum (SR) (blue) and the conventional spectrum with apodization (conv + apo) (black). The signal-to-noise ratio (SNR) was determined using the TopSpin sino routine where the SNR is given by the ratio of the peak intensity and the root mean square deviations (rmsd) in a specified noise region on a 1D trace of isolated signals. The noise and signal regions were chosen consistently for all spectra. c) The linewidth for multiple peaks are plotted. Each circle represents a single peak, horizontal lines indicate the mean and vertical bars indicate the standard deviation where *n* = 73 resolved peaks for each spectrum

As the DNS acquisition method applies to the time period over which the FID is acquired, thus extending the apparent T_2_*, the method should be independent of MAS, offering the same factor 2 resolution gain at fast and ultra-fast MAS speeds. To test this, we acquired an SR-hCH spectrum of AP205 at 50 kHz and measured the linewidth benefits of the ^13^C resonances in the indirect dimension compared to the conventionally acquired hCH spectrum. We observed the same factor 2 resolution improvement in the ^13^C spectrum, even at 50 kHz, in which the linewidth is increasingly dominated by inhomogeneous broadening (Fig. 3), demonstrating the benefit of the DNS method at fast MAS. Traditionally, such an improvement in resolution could be obtained by applying apodization through the same resolution-enhancing function (e.g., an exponential-cosine function) during post-acquisition processing to a conventionally acquired dataset (Levitt et al. 1984). We therefore determined the potential benefit of applying apodization experimentally (with DNS acquisition) over post-acquisition apodization by comparing the SR-DARR spectrum with the conventionally acquired DARR spectrum processed with an exponential-cosine function applied to the indirect dimension. We observed the same gain in resolution through post-acquisition apodization compared to DNS acquisition (Fig. 4c). All the resolvable peaks in the conventional spectrum are narrowed by a factor 2. Furthermore, both methods yielded significant losses in spectrum sensitivity due to amplification of noise (Fig. 4b).

However, the signal-to-noise ratio of the spectrum processed with apodization was 71% lower than the unprocessed spectrum while the DNS acquired SR-DARR was 64% lower when equal measurement times are compared (Fig. 4b). This represents a 20% gain in signal-to-noise ratio of DNS acquisition over post-acquisition apodization as expected from theory (Wenchel et al. 2024).

This 20% gain in SNR of the DNS spectrum compared to the digitally apodized spectrum is non-trivial. Quantification of the resolvable peaks in spectra of U-^13^C,^15^N-AP205 revealed 27% and 14% more peaks above the LOD in the 20 ms SR-DARR compared to the conventional DARR and the post-acquisition apodized DARR respectively (Table 1). Here, we consider any peak with an SNR of ≥ 3 to be detectable signal above the LOD and anything below this level is noise, as routinely defined in analytical NMR methods (Lacey et al. 1999, Subramanian and Webb 1998). With the same criteria applied to both the apodized and SR sampled spectrums. In the crowded 500 ms DARR spectra, 309 peaks were lost below the LOD after post-acquisition apodization compared to the SR-DARR (Table 1). On the other hand, in the highly resolved microcrystalline U-^13^C,^15^N-Ubiquitin spectrum, more peaks were detected in the unprocessed conventional spectrum compared to the SR-DARR. However, DNS sampling still yielded 17% more peaks than the post-acquisition apodized spectrum. As microcrystalline U-^13^C,^15^N-Ubiquitin is near perfectly resolved in the conventional spectrum (not resolution limited), the sensitivity reduction in the SR-DARR out weights the resolution gains. However, this is clearly not the case for AP205 in which peak picking is primarily limited by resolution.

**Table 1:** Comparison of the number of picked peaks above the limit of detection between the different acquisition and processing methods of the 20 ms DARR and 500 ms DARR of AP205 and the 20 ms DARR of ubiquitin. Each dataset was acquired for approximately 30 h

Spectra	Conventional Acquisition	Conventional Acquisition with post-acquisition apodization	Super-resolution
20 ms DARR AP205	738	869	1015
500 ms DARR AP205	1058	891	1200
20 ms DARR Ubi	3185	2200	2650

**Fig. 5:**
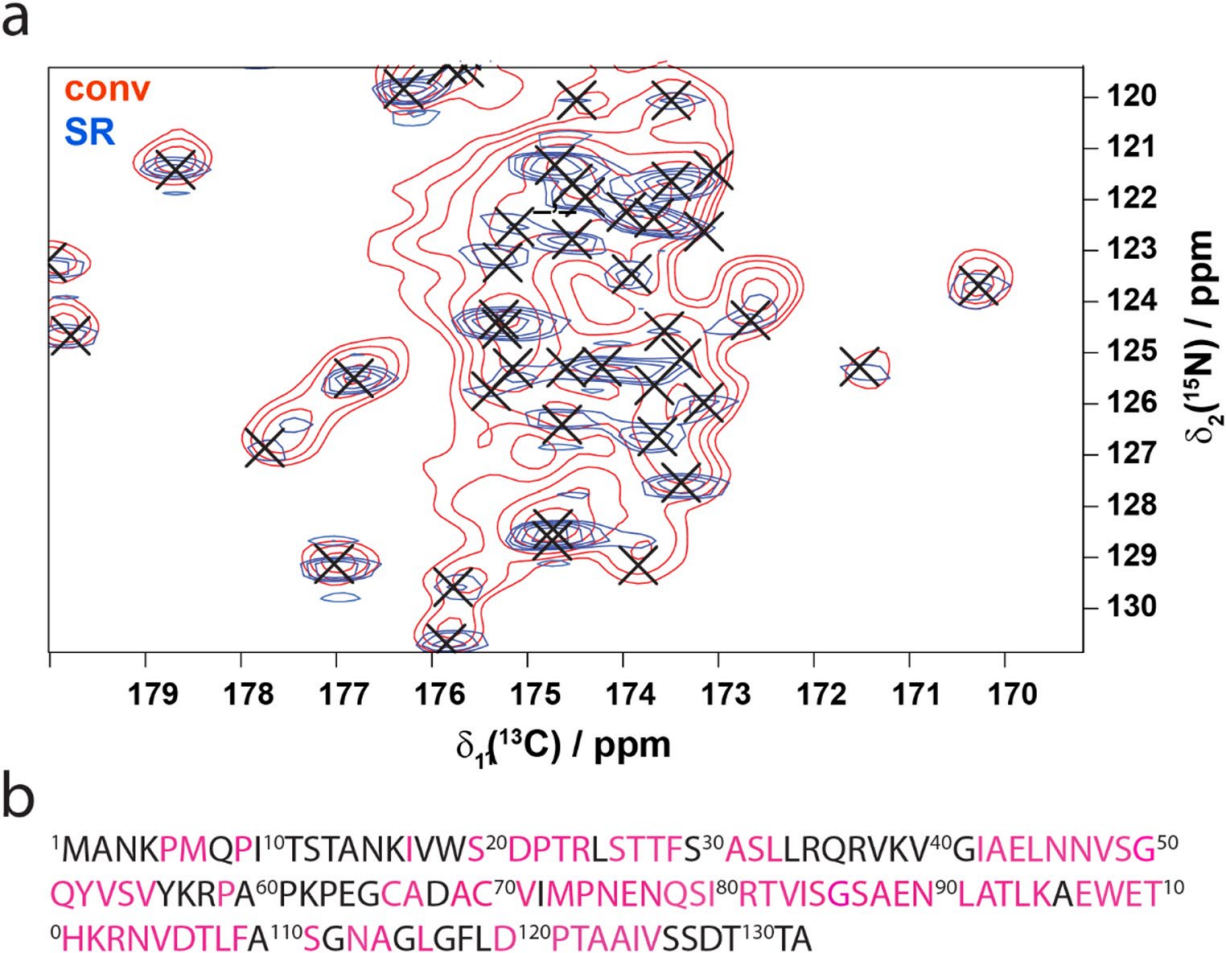
Backbone assignments of AP205. (a) Comparison of conventionally acquired zTEDOR (red) and SR-zTEDOR (blue) with an R_2_ of 475 Hz (151 Hz width). X indicates the position of BMRB assignments for AP205. Data was acquired at 850 MHz with 20 kHz MAS and 1.26 ms heteronuclear mixing time, 792 indirect points at 275 K. (b) Assignments observed (pink) in the SR-DARR spectrum

From the 1200 peaks that were picked in the 500 ms SR-DARR, 1117 could be assigned utilizing the assignments deposited in the BMRB entry: 30094 (Andreas et al. 2016) as our gold-standard combined with the SR-enhanced 20 ms in addition to an SR-enhanced zTEDOR spectrum (Fig. 5a) yielding 62% sequence coverage of the backbone assignments (Fig. 5b) with 56% of all carbon atoms assignable from the BMRB data utilizing three super resolution enhanced 2D datasets (Fig. 5).

Transfer of the BMRB assignments was greatly facilitated by the acquisition of a super resolution enhanced zTEDOR heteronuclear correlation experiment. In fact, the resolution improvement of the SR-zTEDOR was essential in the transfer of many assignments. The zTEDOR collected without DNS acquisition had insufficient resolution in the crowded CO regions to allow for confident assignment (Fig. 5a).

**Fig. 6:**
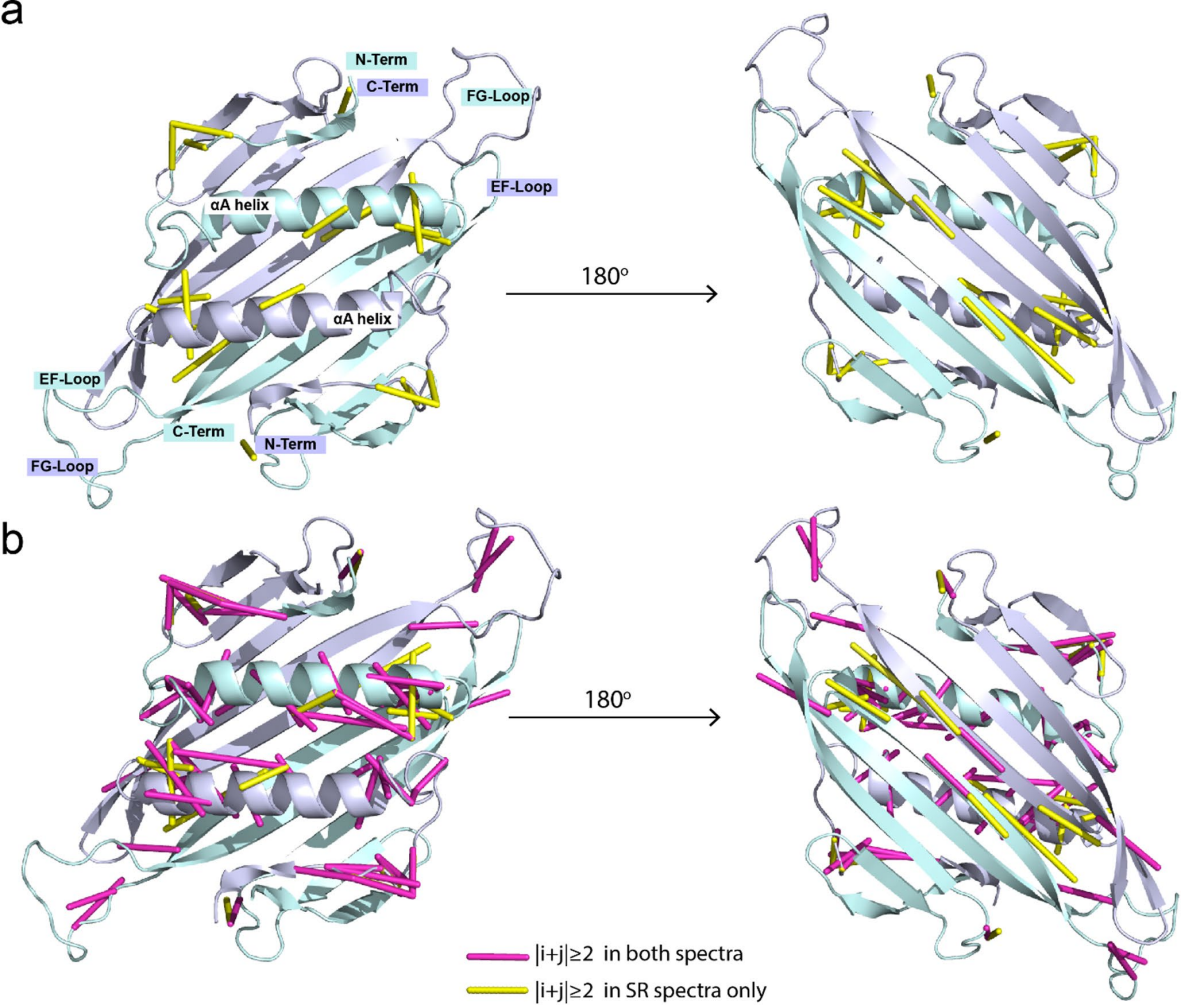
Long-range contacts on the structure of AP205 homodimer observed by super-resolution solid-state spectroscopy. AP205 structure obtained from the PDB ID: 8TW2 (Meng et al. 2024), the two strands which make up the homodimer are colored blue and cyan respectively. (a) $\left|i+j\right|\ge2$ intermolecular contacts observed in the super-resolution spectrum only. (b) $\left|i+j\right|\ge2$ intermolecular contacts observed in both the super-resolution spectrum and the conventional apodized spectrum. Contacts are were determined from peak assignments of 500 ms DARR correlation spectra. Yellow lines indicate $\left|i+j\right|\ge2$ intermolecular contacts observed only in the SR spectrum and magenta lines indicate $\left|i+j\right|\ge2$ intermolecular contacts observed in both the conventional apodized and SR spectra

Of these 1117 peaks, 183 (16.4%) are sequential $\left|i+j\right|=\pm1$ and 44 (3.9%) were long range $\left|i+j\right|\ge2$ contacts (Table 2) as determined from the cryoEM structure as a reference (Meng et al. 2024). In contrast, post-acquisition apodization yielded 36 fewer assigned sequential peaks and 11 fewer assigned long-range contacts (**SI-9**), representing a reduction of 20% of sequential and 25% long-range contacts compared to the DNS sampled spectrum. Mapping of the assigned long-range correlations reveals contacts in the 5–7 Å range most of which are clustered at the N-terminal side of the αA helix and the loop preceding the C-terminal β-strand (Fig. 6). Not all of the peaks in the 500 ms SR-DARR could be assigned from the three 2D datasets, in addition to the observation of additional peaks not reported in the BMRB dataset. Thus, we likely underestimate the total number of possible assignments in the SR spectrum, though we expect the benefit of the SR method over post-acquisition apodization to remain at 30% additional peaks and assignments.

**Table 2:** Breakdown of 1117 assignments made in the 500 ms DARR

Experiment	Intra-residue	sequential	> ± 2
SR-DARR	890	183	44
DARR*	577	147	33

* conventionally acquired DARR spectrum with post-acquisition apodization

## Discussion

We have shown that the super-resolution method employing dynamic number of scans sampling is applicable to solid-state NMR experiments, offering the same factor 2 gain in peak resolution as observed in solution-state. Furthermore, we have shown for a biologically relevant protein system that there is a 20% sensitivity gain over post-acquisition apodization methods, increasing the number of peaks resolved by 15% and affording 20% additional gains in long range contacts that contribute to unambiguous peak assignments and additional 25% gain in long-range contacts that help to constrain tertiary structure towards structural studies.

The resolution gain of factor 2 is on the order of magnitude of that achieved with hahn-echo assisted modulation (HEAD) (Perras 2024, Perras et al. 2025) and deuteration (Xue et al. 2017, Akbey et al. 2010). However, the resolution of the ^13^C dimension benefits more significantly from DNS sampling than the methods mentioned. Particularly as the benefit of deuteration to improving ^13^C linewidths is controversial (Tang et al. 2010, Varga et al. 2007, Morcombe et al. 2005), likely due to sample and labeling scheme dependencies. In addition, the HEAD method, targeting the reduction of the homogeneous component of the linewidth, demonstrates reduced gains in the ^13^C dimension at fast MAS as the homogeneous contribution is reduced by sample spinning (Perras et al. 2025). While DNS sampling appears independent on MAS, at least up to 50 kHz, offering the same resolution gains in the heteronuclear dimension under fast MAS. However, DNS acquisition is limited by its implementation being restricted to the indirect dimension, thus poor resolution in the direct dimension, particularly for ^1^H detected fast MAS experiments may still be challenging unless combined with methods such as selective deuteration or apodization (Levitt et al. 1984). Certainly, none of the methods described achieve the same resolution gains of the machine learning based pure isotropic shift method described by Moutzouri P et al. (Moutzouri et al. 2021, Moutzouri et al. 2023), which remains the superior method for maximum resolution gains, particularly for ^1^H detected spectra. However, the need for acquiring multiple MAS datasets and limited accessibility to the required machine learning based processing tools limits the broader application of this method, particularly to more complex biomolecular samples for structure determination studies. Furthermore, in contrast to the SR method the machine learning based pure isotropic shift method may hallucinate, especially when extended beyond its training domain, yielding non-existing cross peaks or removing cross peaks which may be detrimental for sequential assignment and structure determination. This would be particularly detrimental in situations where one has only a handful to a few dozen long range cross peaks close to the LOD as presented in Table 2. The broad applicability of machine learning approaches will be dependent on experimental validation of hallucination rates and conditions, which can only really be known when a large enough NMR dataset is available to validate against. In this case, more widely applicable experimental gains in spectral resolution such as from SR sampling become an important solution even to this problem.

While DNS acquisition offers one of the simplest spectroscopic implementations for improved resolution, a critical parameter for implementing DNS acquisition is the need to estimate a global R₂ or peak width a priori to define the sampling schedule. This can be challenging in spectrally crowded regions where individual peaks are not resolved in 1D. Acquisition of a 2D DARR or any other reference experiment is optimal for determining the proper R_2_ value for the dataset. As the linewidth can be readily determined from the high-resolution direct dimension, at least in the homonuclear case, it is not necessary to acquire a reference spectrum with high resolution in the indirect dimension.

The implication here is also that there will be some variation in the precise linewidth reduction to different peaks in that peaks with much larger R_2_ values than the estimated R_2_ will be increasingly impacted by sensitivity losses due to noise sampling and are more likely to disappear from the spectrum than peaks within the R_2_ estimate. On the other hand, peaks with ≥ 3 times smaller R_2_ values than the estimated R_2_ are the most likely to contribute to spectrum artifacts through sinc function oscillations in the indirect dimension. Though we did not observe this in AP205, for microcrystalline ubiquitin, such artifacts could be observed on some peaks, particularly those of high intensity along the diagonal. We show that over estimating R_2_ by 2-fold has little impact on the spectrum, suggesting that a sample which exhibits a broad range of T_2_* values will be well tolerated by the SR method. This may in part be due to the use of cross-polarization to generate the initial ^13^C magnetization, which in itself acts as a relaxation filter for extreme outliers. The range of published linewidths measured for ubiquitin at 93 kHz is 80–300 Hz, with a mean linewidth of 137 Hz (Penzel et al. 2019). If we were to use this mean value to calculate a corresponding vc list, the largest linewidth is 2 times the mean and the smallest is 1.7 times less than the mean, well within the SR methods tolerance to R_2_ miss setting. We see this also for proteins like SH3 in which setting the R_2_ value to be the mean of the measured T_2_ values (~ 14.8 ms), places all but two resonances within a value of +/- 2.5 times the mean (Xue et al. 2018).

However, acquisition in the indirect dimension was additionally limited by the J_CC_ coupling and ^1^H-decoupling times. Acquisition times beyond 14 ms, on the order of the 35–38 Hz J_CC_ coupling are expected to have reduced resolution gains since peaks begin to split due to the sampling of the J oscillation. Similar problems have been encountered with the solution-state implementation of DNS acquisition (Gampp et al. 2025). This was overcome by an additional modification to the pulse program and a different weighting function for determining the number of scans. Pseudo decoupling is achieved by skipping over the fid points which approach the zero crossing at 1/2J (Gampp et al. 2025). Although this method is selective for specific couplings, as is the case for many J-decoupling sequences including S^3^E (Laage et al. 2009), LowBASHD (Struppe et al. 2013), band-selective inversions (Zhou et al. 2006, Igumenova and McDermott et al. 2003, Straus 1996) and constant time implementations (Chen et al. 2007), the resolution improvement expected from combined J_COCα_ decoupling and DNS sampling are expected to be significant. However, broadband homonuclear J decoupling in the solid-state remains challenging. The strong J_COCα_ couplings limit SR-indirect acquisition time to 1/2J = 9.1 ms. Acquisition beyond this point broadens the linewidth due to the J-oscillation. Weaker couplings then limit acquisition times to J_CαCβ_ couplings (1/2J = 13 ms) and J_CβCγ_ (1/2J = 14 ms). Constant time approaches can provide a larger bandwidth of J_CC_ decoupling at the expense of additional SNR losses due to T_2_ decay during the constant time period, even in solution (Waudby and Christodoulou 2020), exacerbated in solid samples with typically shorter T_2_ values. Combining pseudo decoupling of J_CαCβ_ as described by Gampp et al. (Gampp et al. 2025) and Waudby et al. (Waudby and Christodoulou 2020) with band-selective J_COCα_ decoupling through selective inversions might be the way forward towards extending SR acquisition times for ultra-fast MAS applications in which the sample T_2_* is more likely to exceed 1/2J (Penzel et al. 2019). Thus, efficient J_COCα_ decoupling has the largest immediate impact on extending acquisition times for larger resolution gains, and in many cases may be sufficient, although the acquisition time will still be constrained by the requirement for strong ^1^H decoupling, at least at slow to moderate MAS frequencies. In this case, conventional acquisition followed by digital apodization may be preferred. However, the vast majority of non-crystalline biologically interesting samples are unlikely to have such long T_2_* decay values due to increased heterogeneity and dynamics, at least under moderate MAS. However, this does have implications for the combination of deuteration methods and ultra-fast MAS with DNS acquisition in which T_2_ relaxation rates will lengthen (Tang et al. 2010, Penzel et al. 2019), increasing the need for implementation of DNS to acquisition times that exceed 1/2J. Ultra-fast MAS also greatly reduces instrumentation limitations imposed by strong ^1^H decoupling, making DNS sampling practical for samples with very long T_2_* values.

The doubling in effective resolution directly translates to more peaks that can be picked and therefore assigned. We could confidently transfer 62% more assignments from the BMRB onto the short range 20 ms SR-DARR compared to conventionally acquired spectrum as a direct result of the resolution increase. Post-acquisition apodization of the conventional spectrum reduces the number of resolvable peaks by another 15% compared to the SR-DARR as a result of SNR losses. We attribute the improved sensitivity of DNS to reduced noise variance (σ^2^) at longer increments where σ²(t_late) = σ₀²/NS(t) << σ_uniform, while digitally apodized spectra pass through the full pre-existing thermal noise at late points where the apodization function h(t) ≈ 1, yielding higher noise variance at later increments than in DNS sampled FIDs. The data demonstrates the significant increase in the information extracted from 2D ^13^C-^13^C datasets at moderate MAS frequencies, when resolution is the limiting factor. This is despite the sensitivity losses incurred by employing DNS acquisition. Of course, this is not the case when resolution is not the limiting factor, as is observed for the highly resolved microcrystalline ubiquitin sample.

The super-resolution spectrum resolved approximately 1200 peaks, a significant increase over the conventional acquisition. Of these 1200 peaks, 1117 could be successfully assigned utilizing data from the BMRB with 61% of the backbone resonances assignable and 56% of all carbon atom assignments. We were unable to confidently assign 92 peaks from the 500 ms SR-DARR, which would require the acquisition of at least one additional 3D experiment. It is therefore likely that we underestimate the true benefit of the SR method for obtaining assignments and restraint data for structural studies. Furthermore, a larger proportion of the peaks lost in the conventionally acquired spectrum are long-range contacts, crucial information for constraining tertiary structures during structure calculation protocols. Many of these contacts were also $\left|i+j\right|\pm2$, valuable contacts for reducing ambiguity in the assignment process. Thus, there is clear value in implementing DNS acquisition over post-acquisition processing for solid-state NMR structural studies. The resolution gain also improves data quality, making the data amenable to automated peak picking and assignment tools.

DNS is a type of non-uniform sampling. A number of NUS implementations exist but can be broadly categorized into on grid and off grid schedules. While off grid schedules require more complex FID reconstruction methods prior to Fourier transform, and thus the reduction of spectrum artifacts is non-trivial, on grid schedules are more common and offer more straight forward processing and are comparable to DNS sampling (Mobli and Hoch 2014). On grid NUS typically yields a 1.2–2.2 gain in SNR, comparable to the 1.2 SNR gain we observe in the DNS acquisition, albeit on the lower end (Rovnyak et al. 2011, Paramasivam 2012, Kaur et al. 2020). Accordingly for NUS sampling schemes in general, the NUS sensitivity theorem proposed by Palmer et al. states that any decreasing sampling density applied to any exponentially decaying signal always results in higher SNR per √time than uniform sampling (Palmer et al. 2015), particularly if sampling is T_2_ matched (Penzel et al. 2019). Furthermore, weighted averaging has been described by Waudby et al. (Waudby and Christodoulou et al. 2012), Qiang (2011) and Kumar et al. (1991). Weighted averaging with T_2_* matched exponential decay functions reproduces the sensitivity gains afforded by similar NUS sampling schemes without improving resolution of the indirect dimension (Qiang et al. 2011, Kumar et al. 1991). Only averaging schemes weighted with exponential growth functions afford the resolution improvements we observe (Waudby and Christodoulou 2020). Multiplication of this weighting scheme with a cosine function improves the practicality of the SR method at longer acquisition times in which the number of scans required at later increments becomes prohibitively large. This does however, come at the expense of compatibility of the SR FID with linear prediction methods, unlike the averaging scheme weighted only by an exponential growth function (Waudby and Christodoulou 2012).

Direct comparison between the benefits of the SR-2D over 3D or NUS sampled 3D acquisition is difficult, as the additional coherence transfer step in 3D experiments increases the information content of the resultant spectrum, despite the much courser resolution in each of these dimensions. The ‘resolving power’ of a 3D experiment is gained through frequency dispersion and not through improved peak resolution. However, NUS enables time-equivalent higher-dimensional experiments without resolution gains per se (unless increased sampling beyond the uniformly sampled t_max_), trading ~ 10–20× coarser peak resolution per indirect dimension (when comparing a typical 3D of 64 indirect complex points to the 1216 complex points acquired in the SR-2D). Thus, we do not view SR-2D as a replacement for 3D spectroscopy but as complementary spectra that can significantly increase the number of contacts for structure determination studies. Beyond the identification of long-range distance restraints, improved effective resolution is also beneficial for chemical shift perturbation (CSP) analysis, in solid-state NMR, particularly in cases of weak hydrogen bonding in heterogeneous environments where the CSP is comparable to or smaller than the linewidth (Sabena et al. 2026). The precision with which resonance frequencies can be determined is fundamentally limited by the signal-to-noise ratio and the effective observation time of the free induction decay, as described by the Cramér–Rao lower bound for exponentially damped sinusoids in noise (Steven and Kay 1993). By reducing noise variance at longer evolution times, the DNS approach extends the effective detectable coherence lifetime without modifying the intrinsic signal envelope. This increases the usable observation time and thereby improves frequency estimation precision. In crowded solid-state spectra, where small CSPs are often obscured by peak overlap and limited frequency precision, this enhanced effective resolution can facilitate more reliable detection of subtle chemical shift changes associated with ligand binding, conformational rearrangements, or polymorphism.

## Conclusion

We have demonstrated the successful application of dynamic number of scan sampling in the solid-state, achieving a factor 2 gain in resolution in crystalline and non-crystalline biomolecular samples. We additionally demonstrate sensitivity gains of DNS acquisition over post-acquisition apodization methods that yields 20% more resolved peaks and 25% more long-range contacts for unambiguous resonance assignments and distance restraints. Thus, DNS acquisition increases the information extracted from 2D datasets. We hope that such a method will contribute to reducing the need for multiple 3D experiments for biomolecular structure determination in order to improve the accessibility and feasibility of solid-state NMR for structural studies.

## Materials and methods

### Protein expression and purification

#### AP205

Uniformly labeled ^13^C, ^15^N AP205 was prepared as previously described (Radiom et al. 2023). Briefly, protein expression was performed in BL21 (DE3) *Escherichia coli* cells containing the AP205 gene on a pTK190 vector and induced with 1 mM IPTG after reaching an OD_600_ of 0.5 in M9 Minimal Medium. The cells were incubated overnight at 28 °C after which they were lysed via sonication. AP205 was purified from the lysate with 2 mM NiSO_4_, the resulting pellet was washed with 50 mM Tris pH 7.5, 150 mM NaCl buffer (TBS buffer) and resuspended 1 mL of TBS buffer and 6 mM EDTA. Lipid was removed with the addition of 2% of final volume of Triton X-100 and allowed to phase-separate at 42 °C for 20 min. The sample was spun down and the lipid pellet discarded. AP205 was precipitated with the addition of 7 mM NiSO_4,_ once again washed with TBS buffer and resuspended with 20 mM EDTA. The pellet was resuspended gently overnight by shaking at 4 °C. The protein solution was sedimented into a zirconia 3.2 mm–1.3 mm MAS-NMR rotor for 16 h at 4 °C at 210,000 × g using custom-made filling tools.

#### Ubiquitin.

Uniformly ^13^C and ^15^N labeled ubiquitin was expressed and purified as described previously (Sass et al. 1999). The sitting drop method described by Igumenova et al. (2004). was used to obtain microcrystals of the protein. The crystals were harvested after 10–14 days of being incubated at 4 °C in the precipitation buffer (3:2 volume ratio of 2-methylpentanediol-2,4 and citrate buffer at pH 4.1 and 0.05% sodium azide) and centrifuged into 3.2 and 1.3 mm MAS-NMR rotors.

### Solid-state NMR experiments

Solid-state NMR experiments were performed on a wide-bore 850 MHz Bruker Avance NEO and a standard-bore 1200 MHz Bruker Avance NEO (TopSpin 4) spectrometers. ^1^H-detected spectra were acquired using a commercial 1.3 mm triple resonance MAS probe (Bruker Biospin) at 50 kHz MAS at 265 K hCH experiment was acquired with tangential ramp cross-polarization and 40 kHz spin-locking field on ^13^C and 20 kHz WALTZ16 ^13^C decoupling during acquisition.^13^C-detected DARR spectra were acquired using 3.2 mm rotors in a commercial E-FREE triple resonance probe (Bruker Biospin) at 17 kHz for Ubiquitin and 20 kHz for AP205. All experiments were conducted at 275 K with linear ramp cross polarization with 50 kHz ^13^C spin-locking field and 96 kHz SPINAL64 ^1^H decoupling. zTEDOR spectra were acquired with 35 kHz ^15^N spin-locking field and 1.26 ms TEDOR mixing. All spectra were processed in TopSpin 3, additionally PROSA (Güntert et al. 1992) was used for smoothing of super-resolution acquired spectra (Gampp et al. 2024). ^13^C-detected spectra were acquired with 96 kHz SPINAL64 decoupling. Additional acquisition parameters are shown in **SI-10** in the Supplementary Information.

For implementation of DNS acquisition, the number of increments in the indirect dimension are calculated as follows:

2 $${n_{increment}} = 4\left( {\frac{3}{4} \cdot \frac{{2\pi }}{{({R_{enh}})\Delta t}}} \right)$$

Where f is the resolution enhancement factor (0.5 for doubling of the resolution), ∆t is the indirect increment in µs, the multiplication by 4 ensures proper acquisition of complex points, and ${R}_{enh}$ is the enhanced R_2_ value given by:

3 $${R}_{enh}={R}_{2}\mathrm{*}(1-f)$$

And R_2_ is estimated from the peak width in Hz as follows:

4 $${R}_{2}=\pi\left(FWHH\right)$$

The number of scans per increment is then calculated by:

5 $$ns=\left({N}_{1}{e}^{{R}_{enh}t}\mathrm{cos}\left({\omega}_{apo}t\right)\right)$$

Where N_1_ is the starting number of scans (typically 4–8), and ${\omega}_{apo}$ is the apodization frequency given by:

6 $${\omega}_{apo}=\frac{\pi}{2}\frac{1}{\left({n}_{inc}-1\right){\Delta}t}$$

### Data processing and analysis

All spectra were processed in TopSpin 4.0. The post acquisition apodization was implemented using PROSA. Using the apodization function described in **SI-5**. Linewidths were determined from 1D slices by taking the peak width at half height. The signal-to-noise ratio of spectra was also taken from 1D slices using the sino function within TopSpin. The limit of detection (LOD) was chosen as sino = 3 (Lacey et al. 1999).

Spectrum assignments and peak picking was done in ccpnmr.v3 (Skinner et al. 2016).

## Supplementary Information

Below is the link to the electronic supplementary material.

Supplementary Material 1

## Data Availability

NMR data produced in this work are available upon request.
