# Supplementary material for: Super-Resolution solid-state NMR Spectroscopy
Source: J Biomol NMR. 2026 Mar 31;80(1):11. doi: 10.1007/s10858-026-00490-5 (PMC13038466; doi:10.1007/s10858-026-00490-5)
Supplement: Supplementary file 1 — Supplementary Material 1 [file 10858_2026_490_MOESM1_ESM.docx]

**Supporting Information**

**Super-Resolution solid-state NMR Spectroscopy**

Olivia Gampp, Riccardo Cadalbert Roland Riek* and Sarah A Overall*

Institute of Molecular Physical Science, ETH Zürich, Vladimir-Prelog-Weg 2, CH-8093 Zürich, Switzerland

* to whom correspondence should be addressed to: [sarah.overall@phys.chem.ethz.ch](mailto:sarah.overall@phys.chem.ethz.ch), [roland.riek@phys.chem.ethz.ch](mailto:roland.riek@phys.chem.ethz.ch)

**Table of Contents**

**SI-1:** Estimating T_2_ ………………..…………………………………………………………………....2

**SI-2**: Python script for generation of increment list………………………………………………….....3

**SI-3**: Pulse code for implementation of Super Resolution DARR ……………………………………..4

**SI-4**: Spectra acquired with incorrect R_2_ estimate…………………………………………………........6

**SI-5**: Effect of R_2_ estimate on peak fidelity and linewidth……………………………………………...7

**SI-6**: FID and spectral artifacts due to DNS acquisition removed by FID smoothing………………….8

**SI-7**: Function to smooth FID…………………………………………………………………………...9

**SI-8**: Python version of the PROSA smoothing function……………………………………………...10

**SI-9**: $\left| i+j \right|\geq2$ intermolecular contats sobserved in both SR and conventionally apodized spectra...12

**SI-10**: Acquisition parameters…………………………………………………………..…………......13


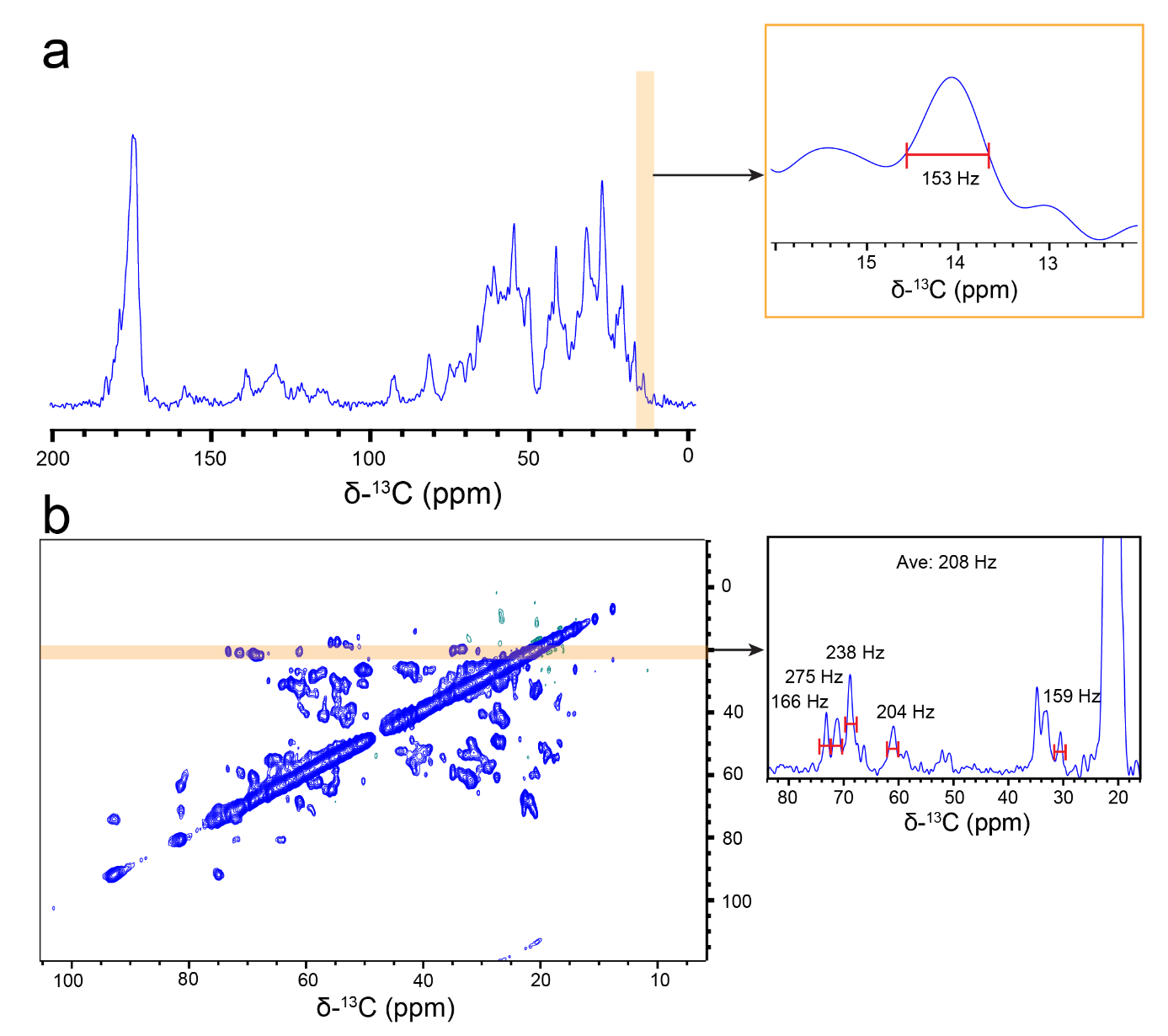


**SI-1** Estimating T_2_. a) Estimates of peak linewidth can be done in 1D using resolved peaks, particularly in the isoleucine Cd region. From this we could estimate a peak width of 153 Hz. b) Alternatively, and more accurately, a 2D-DARR can be used to estimate the linewidth in the direct dimension. Sufficient increments in the indirect dimension are needed to obtain sufficient resolution. The DARR shown here was acquired with 4 scans, 256 increments in the indirect dimension and 2048 points in the direct dimension. The orange box indicates the region of the spectrum from which the linewidth can be estimated. A 1D slice through this region is shown with the measured peak widths given and the average peak of 208 Hz. This level of accuracy is sufficient for calculating the variable counter list and obtaining an artifact free SR-DARR spectrum.

**SI-2**: Python script for generation of increment list

import numpy as np

from math import pi

#definitions

R_2 =1050 # define relaxation rate in Hz

f = 0.5 # factor with by which the peak-width should be reduced

dt = 14.7 * 1e-6 # dwell time in s

N_1 = 4 # starting number of scans

cl = 4 # phase cycle length

# calculate the optimal number of time increments for the cosine-modulated method

# and round it up to a multiple of 4 (States-TPPI)

#########

n_inc = int(3 / 4 * 2 * pi / (R_2 * (1-f) * dt))

n_inc += n_inc % 4

#########

#generate vc_list SR#

#########

omega_apo = pi / 2 * 1 / ((n_inc - 1) * dt)

t = np.arange(0, n_inc) * dt

#calculate the number of scans at every increment (already rounded down to an integer value)

NS = np.floor(N_1 * np.exp(R_2 * f * t) * np.cos(omega_apo * t)).astype(int)

#adjust NS so that it is compatible with the phase cycling

r_N = NS % cl

NS -= r_N

# make sure that there are no negative number of scans

NS_SR = np.maximum(NS, 0)

#########

#NS_SR is 100% dynamic number of scans that should be acquired for SR

#Once generated, copy the vc list into the following TopSpin version #folder: TopSpin3.8.0/exp/data/stan/nmr/lists/vc

#The list is available under the ‘vc list’ option in the acquisition #parameters.

**SI-3**: Pulse code for implementation of Super Resolution DARR. Changes to the regular DARR pulse sequence are highlighted in blue text.

;2D DARR exchange experiment with CP

;phase-sensitive detection in t1 by States-TPPI

;minimal offset- and quad-compensated phase cycle

;

;pl11 : X 90 power level

;pl1 : X CP power level

;pl2 : H 90 degree power level

;pl10 : H adiabatic sweep power level (CP)

;pl12 : decoupling power level

;pl14 : H recoupling power level (equal to the MAS)

;p14 : H recoupling pulse

;p1 : X 90 degree pulse

;p3 : H 90 degree pulse (not used)

;p15 : CP contact

;p31 : TPPM pulse

;d3 : dead time delay

;l18 : dummy scans

;l7 : super res counter calculated from VC list

;l3 : TD in the indirect dimension: calculated from td1

;States-tppi : acquisition mode

;$OWNER=ciwa

#include <Avance.incl>

#include <trigg.incl>

"acqt0=1u*cnst11"

"d15=td*dw"

"in0=inf1"

"d0=0.1u"

"p4=p1"

define list<loopcounter> SUPER=<$VCLIST> ; SUPER RES

"l3= td1/2" ; SUPER RES

1 ze

;--------Begin Dummy Scans-------

2 1m do:f2

if "(2*p1+p3+p15+d0+d3+(td*dw))>800ms" goto 8

d1

trigg

1u fq=cnst22:f1

(p2 pl2 ph1):f2

(p15 pl1 ph5):f1 (p15:sp0 ph0):f2

0.1u fq=0:f1

(d0 cpds2):f2

0.1u fq=cnst1:f1

(p1 pl11 ph2):f1 (1u do):f2

; d14 ;use if PDSD is preferred (removing the next line)

(p14 pl14 ph0):f2

(p4 pl11 ph4):f1 (1u pl12):f2

0.1u

0.1u cpds2:f2

d15 ; d15 is calculated to be equal to the acquisition time taking

; acquisition decoupling into account

1u do:f2

lo to 2 times l18 ; l18 determines the number of dummy scans.

;-------End Dummy Scans-----

3 10u do:f2

4 10u do:f2

5 1m do:f2

if "(2*p1+p3+p15+d0+d3+(td*dw))>800ms" goto 8

d1

"l7 = SUPER[l8]" ;Implementation of the vc list for DNS acquisition

trigg

1u fq=cnst22:f1

(p2 pl2 ph1):f2 ;90 pulse on proton

(p15 pl1 ph5):f1 (p15:sp0 ph0):f2 ;CP to carbon

0.1u fq=0:f1

(d0 cpds2):f2 ;t2 evolution

0.1u fq=cnst1:f1

(p1 pl11 ph2):f1 (1u do):f2 ;z-storage pulse

;d14 ;use if PDSD is preferred (removing the next line)

(p14 pl14 ph0):f2 ;DARR proton recoupling

(p4 pl11 ph4):f1 (1u pl12):f2 ; readout pulse

0.1u

goscnp ph31 cpds2:f2 finally do:f2 ; fid acquisition

3m ipp1 ipp2 ipp4 ipp31

lo to 3 times l7 ;super-resolution counter

100u do:f2 wr #0 if #0 ze dp2

lo to 4 times 2 ;ensures complex points acquisition

10u id0

10u iu8

lo to 5 times l3

8 1u do:f2

exit

;--------Phase Cycles----------

ph1 = 1 3 ;H 90

ph0 = 0 ;H CP

ph5 = 0 ;X CP

ph2 = 1 1 3 3 ;3 1 1 3 ;X storage

ph4 = 3 3 3 3 ;1 1 1 1 0 0 0 0 2 2 2 2 ;X readout

ph31 =0 2 2 0 ;2 0 0 2 1 3 3 1 3 1 1 3 ;receiver phase

**
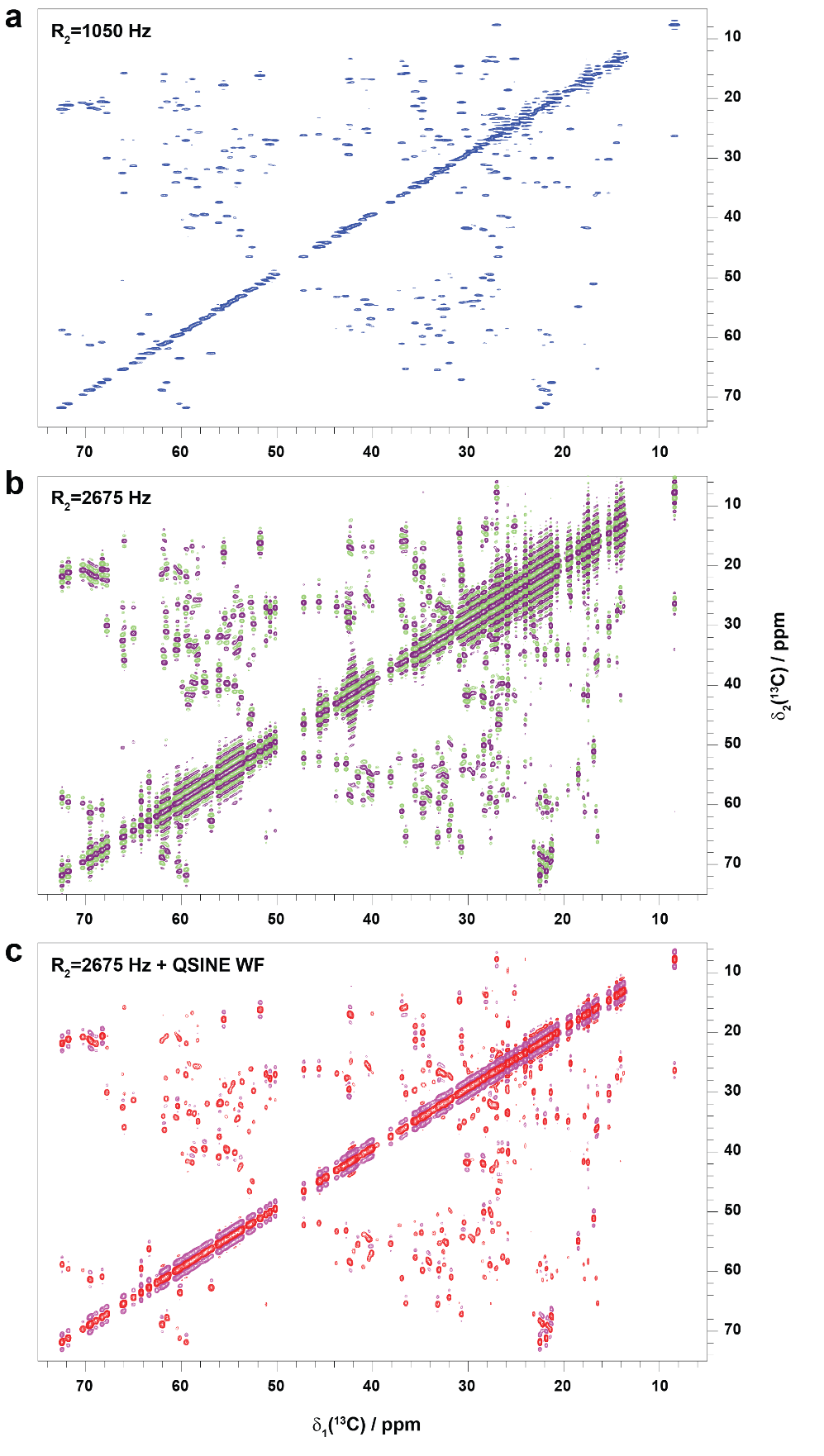
**

**SI-4**: Spectra acquired with incorrect R_2_ estimate. SR-DARR of U-13C,15N-Ubiquitin acquired with a) an R_2_ of 1050 Hz (FWHH = 334 Hz), b) an R_2_ of 2675 Hz (FWHH = 851 Hz) and c) an R_2_ of 2675 Hz (FWHH = 851 Hz) where the spectra is additionally processed with a QSINE (SSB= 3) function prior to Fourier Transform.


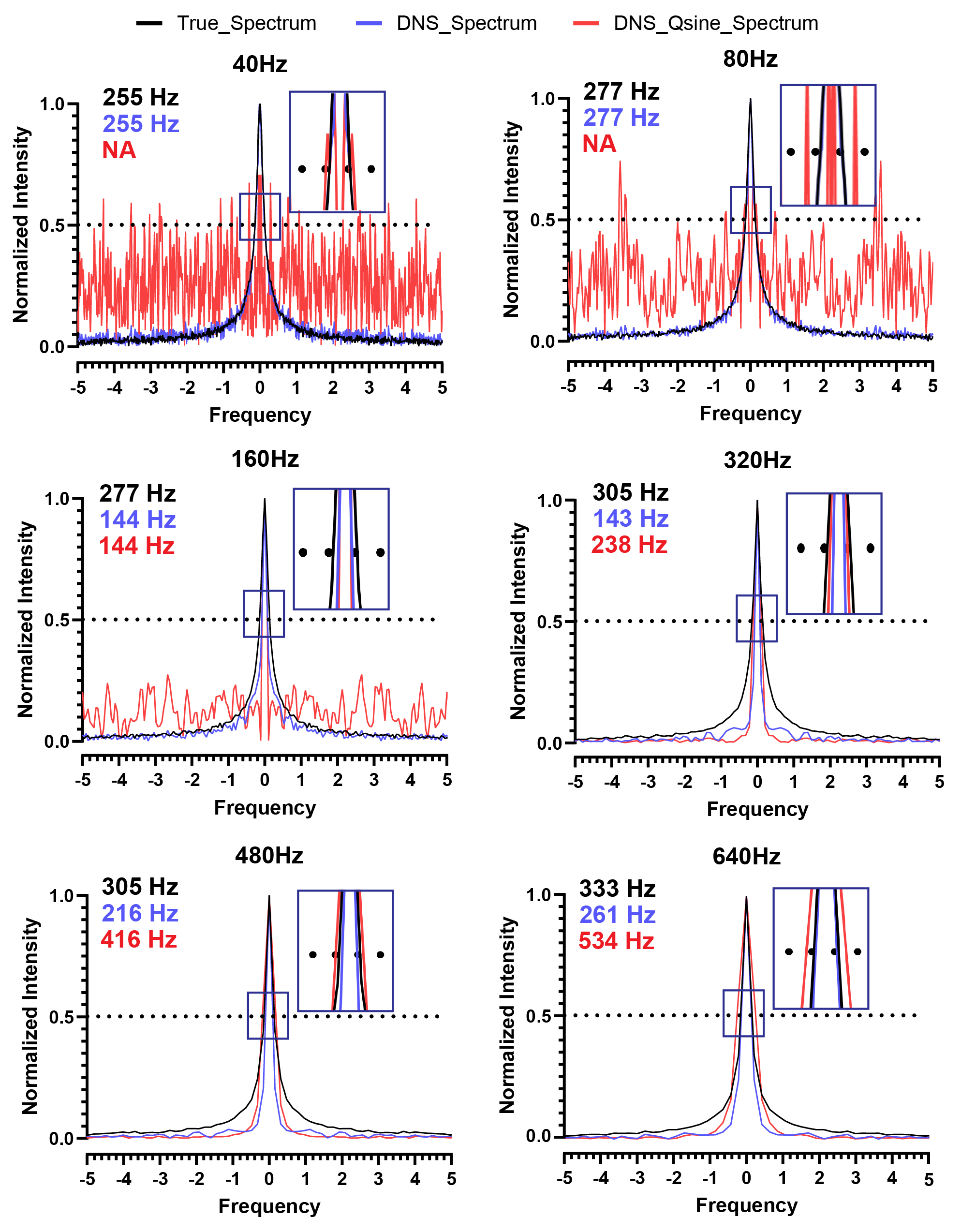


**SI-5**: Effect of R_2_ estimate on peak fidelity and linewidth. Spectra were simulated with MATLAB code simulating a conventional spectrum (black line), DNS sampled spectrum (blue line) and qsine compensated DNS spectrum (red line). Spectra are Fourier transformed with 4*td zero filling. The conventional spectrum is simulated with a T_2_* of 1.98 ms (160 Hz FWHH). DNS spectra are simulated by applying the DNS sampling scheme over the conventional FID (with the DNS sampling scheme determined as described in SI-2). A noise estimate of 0.05 is added to the spectra, the intensities are normalised and the linewidth is determined as the width at half-height of the simulated peaks. Qsine window function was applied with SSB = 3. Spectra are processed using the number of points determined by the DNS vc script in SI-2 (as would be the case experimentally).


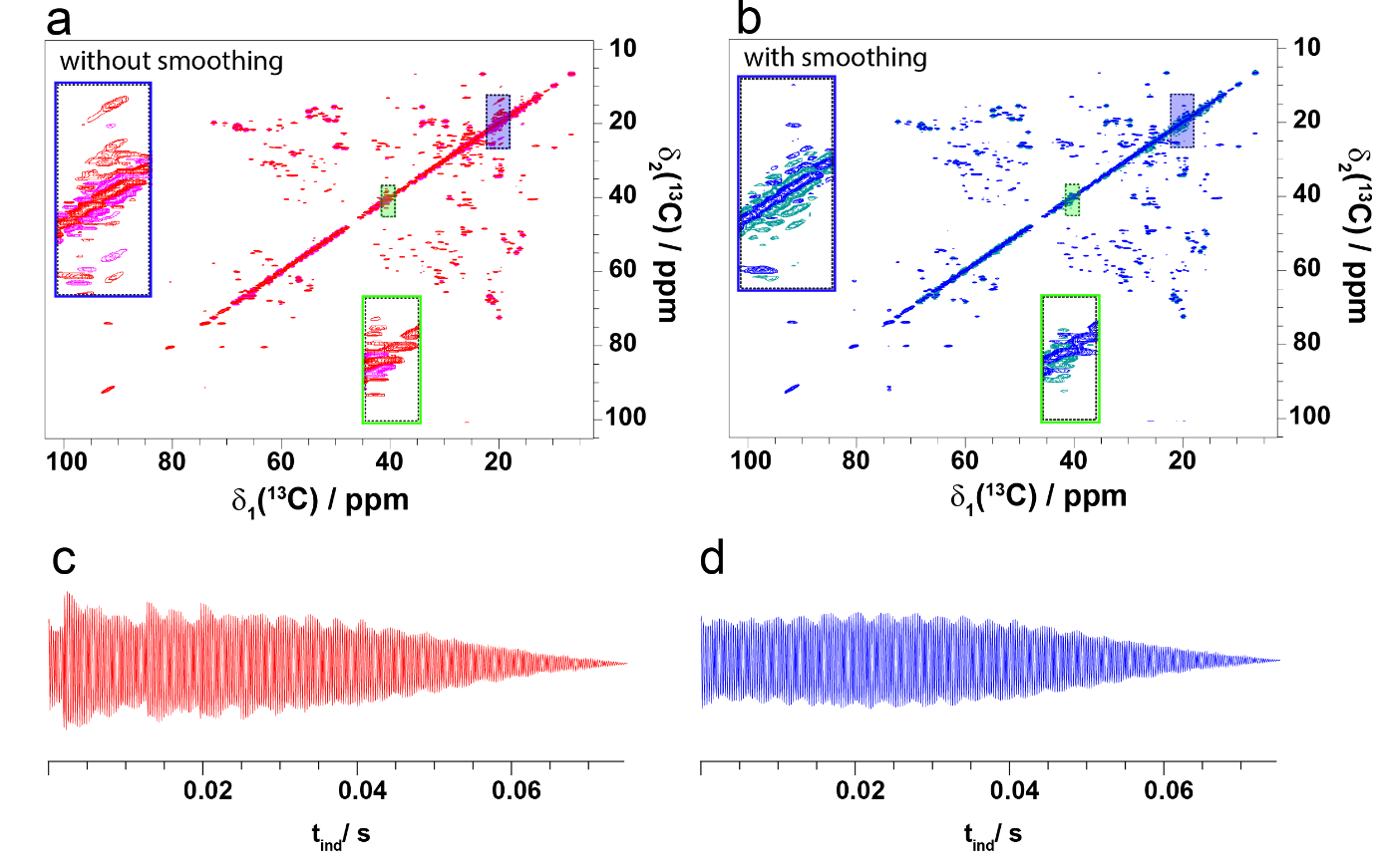


**SI-6**: FID and spectral artifacts observed due to the DNS acquisition. a) 20 ms SR-DARR with no FID smoothing and processed just with TopSpin. The boxes indicate regions where peak lobes are observed. b) 20 ms SR-DARR with FID smoothing. c) FID before smoothing. d) The same FID but smoothed with PROSA.

**SI-7**: Function to smooth FID

dimension 2

R_2 = 1050.0

f = 0.5

dt = 14.7E-6

N_1 = 4

cl = 4

n_inc = int(3.0 / 4 * 2 * pi / (R_2 * (1-f) * dt))

n_inc = n_inc + mod(n_inc,4)

omega_apo = pi / 2 * 1 / ((n_inc - 1) * dt)

do i 0 n_inc-1

t = i * dt # dt is dwell time in seconds

#calculate the NS that were acquired

N_scans = int(N_1*exp(R_2*f*t)*cos(omega_apo*t))

r_N = mod(N_scans,cl)

#if (r_N.eq.cl) r_N=0

N_scans = N_scans - r_N

#calculate the continuous number of scans

N_cont = N_1 * exp(R_2 * f * t) * cos(omega_apo * t)

if (N_scans.gt.0) then

#smoothing value N

N = N_cont/N_scans

else

N=1.0

end if

multiply N i+1

end do

dimension 1 2

**SI-8:** Python version of the PROSA smoothing routine (Bruker SER compatible)

#!/usr/bin/env python3

import numpy as np
from math import pi, exp, cos

# -------- USER PARAMETERS --------
ser_in = "ser"
ser_out = "ser_smoothed"

R_2 = 1050.0
f = 0.5 #enhancement factor
dt = 14.7e-6 #increment
N_1 = 4 #NS
cl = 4 #phase cycle

# direct dimension size (points per FID)
TD2 = 2048 # change if needed
# ---------------------------------

# ---- calculate increments ----
n_inc = int((3.0/4.0) * 2*pi / (R_2*(1-f)*dt))
n_inc = n_inc + (n_inc % 4)

omega_apo = (pi/2.0) / ((n_inc - 1) * dt)

# ---- smoothing factors ----
smoothing = np.ones(n_inc)

for i in range(n_inc):

t = i * dt

N_scans = int(N_1 * exp(R_2 * f * t) * cos(omega_apo * t))
r_N = N_scans % cl
N_scans = N_scans - r_N

N_cont = N_1 * exp(R_2 * f * t) * cos(omega_apo * t)

if N_scans > 0:
smoothing[i] = N_cont / N_scans
else:
smoothing[i] = 1.0

# ---- read SER file ----
raw = np.fromfile(ser_in, dtype=np.int32)

# convert interleaved real/imag → complex
raw = raw.reshape(-1,2)
data = raw[:,0] + 1j*raw[:,1]

# reshape to 2D matrix
data = data.reshape(n_inc, TD2)

# ---- apply smoothing (t1 only) ----
data = data * smoothing[:,None]

# ---- convert back to interleaved int32 ----
data_flat = data.reshape(-1)

out = np.zeros(len(data_flat)*2, dtype=np.int32)
out[0::2] = np.real(data_flat)
out[1::2] = np.imag(data_flat)

# ---- write new SER ----
out.tofile(ser_out)

print("Done.")
print("Output written to:", ser_out)
print("Indirect dimension points:", n_inc)

#the new ser_out should be readable by TopSpin if placed in an experiment folder as a normal fid would appear.

| 23.ARG.CB | 25.SER.CA | in both |
| --- | --- | --- |
| 25.SER.CB | 23.ARG.CB | in both |
| 46.ASN.CA | 43.GLU.CG | in both |
| 51.TYR.CA | 49.GLY.CA | in both |
| 57.ARG.CB | 59.ALA.CB | in both |
| 70.VAL.CA | 73.PRO.CA | in both |
| 70.VAL.CGy | 72.MET.CA | in both |
| 75.GLU.CB | 79.ILE.CG2 | SR-only |
| 80.ARG.CB | 78.SER.CB | SR-only |
| 82.VAL.CA | 80.ARG.CA | in both |
| 86.SER.CA | 84.SER.CB | SR-only |
| 87.ALA.CA | 85.GLY.CA | SR-only |
| 90.LEU.CG | 95.ALA.CB | in both |
| 91.ALA.CB | 93.LEU.CDx | in both |
| 92.THR.CA | 94.LYS.CE | SR-only |
| 92.THR.CB | 90.LEU.CB | SR-only |
| 93.LEU.CA | 91.ALA.CB | in both |
| 93.LEU.CG | 91.ALA.CA | in both |
| 94.LYS.CA | 96.GLU.CB | in both |
| 94.LYS.CD | 101.LYS.CB | in both |
| 94.LYS.CE | 96.GLU.CB | in both |
| 95.ALA.CA | 91.ALA.CB | in both |
| 96.GLU.CG | 94.LYS.CE | in both |
| 97.TRP.CB | 99.THR.CB | in both |
| 101.LYS.CG | 98.GLU.CB | SR-only |
| 103.ASN.CA | 101.LYS.CG | in both |
| 108.PHE.CA | 110.SER.CB | in both |
| 109.ALA.CA | 107.LEU.CDy | in both |
| 109.ALA.CB | 111.GLY.CA | in both |
| 111.GLY.CA | 113.ALA.CB | in both |
| 113.ALA.CB | 115.LEU.CA | in both |
| 119.ASP.CA | 121.THR.CB | SR-only |
| 119.ASP.CB | 121.THR.CB | in both |
| 120.PRO.CA | 122.ALA.CB | in both |
| 120.PRO.CA | 122.ALA.CA | SR-only |
| 121.THR.CB | 123.ALA.CA | SR-only |
| 121.THR.CB | 123.ALA.CB | in both |
| 122.ALA.CB | 120.PRO.CG | in both |
| 123.ALA.CA | 121.THR.CB | in both |
| 123.ALA.CA | 121.THR.CG2 | in both |
| 123.ALA.CB | 125.VAL.CA | in both |
| 124.ILE.CA | 122.ALA.CA | in both |
| 124.ILE.CA | 121.THR.CG2 | in both |
| 126.SER.CA | 122.ALA.CA | SR-only |
|  |  |  |

**SI-9**: $\left| i+j \right|\geq2$ intermolecular contacts observed in the SR and conventional apodized spectrum. Contacts observed in both spectra are coloured magenta and contacts observed only in the SR spectrum (yellow). Contacts in white are not displayed in Figure 6 due to overlap with already visualised contacts.

| Experiment  (Sample) | # Scans | Indirect points  (time in ms) | Direct points  (time in ms) | Increment delay  (µs) | | R_2_ estimate  (Hz) | MAS (kHz) | Mixing time  (ms) | Total Experiment time  (h) |
| --- | --- | --- | --- | --- | --- | --- | --- | --- | --- |
| Conv-DARR  (Ubiquitin) | 28 | 1224 (9.0) | 1550 (9.0) | | 14.7 | NA | 17 | 20 | 10 |
| SR-DARR  (Ubiquitin) | N1=4  (28 ave) | 1224 (9.0) | 1550 (9.0) | | 14.7 | 1050 | 17 | 20 | 9 |
| Conv-DARR  (AP205) | 28 | 1216 (8.9) | 1536 (9.0) | | 14.7 | NA | 20 | 20 | 29 |
| SR-DARR  (AP205) | N1=4  (28 ave) | 1216 (8.9) | 1536 (9.0) | | 14.7 | 1050 | 20 | 20 | 29 |
| Conv-DARR  (AP205) | 28 | 1216 (8.9) | 2048 (11.9) | | 14.7 | NA | 20 | 500 | 33 |
| SR-DARR  (AP205) | N1=4  (28 ave) | 1216 (8.9) | 2048 (11.9) | | 14.7 | 1050 | 20 | 500 | 31 |
| SR-TEDOR  (AP205) | N1=8  (56 ave) | 792 (19.8) | 1024 (11.3) | | 50.0 | 475 | 20 | 1.26 | 23 |
| SR-hCH  (AP205) | 4  (28 ave) | 2156 (9.0) | 2142 (9.0) | | 8.33 | 1050 | 50 | 1 | 16 |

**SI-10**: Acquisition parameters for the experiments carried out in the presented study.
